# Retraction of ‘Mutually exclusive splicing regulates the Nav 1.6 sodium channel function through a combinatorial mechanism that involves three distinct splicing regulatory elements and their ligands’

**DOI:** 10.1093/nar/gkad1123

**Published:** 2023-11-21

*Nucleic Acids Research*, Volume 40, Issue 13, 1 July 2012, Pages 6255–6269, https://doi.org/10.1093/nar/gks249

Following allegations of image manipulation in Figures 2B, 2D, 4C, 4E and 6B-D in 2022, the journal conducted a brief investigation, referred the matter to the authors’ institution, and published an Expression of Concern.

The authors have since been unable to produce the original data. Because of the lack of data to support the original conclusions and after an investigation substantiated the issues raised about Figures 2B, 4E, and 6B-D, in September 2023, the institutional committee investigating the allegations recommended retracting the article. The Editors of the journal are now retracting the article based on the following findings of the institutional investigation:

- Figure 2B: Difference analysis software indicated that lanes 1, 3, and 4, are identical.
- Figure 4E: Difference analysis software indicated that lanes 9 and 10 are identical.
- Figure 6B-D: Difference analysis software indicated that lanes or areas within panels have been duplicated within all 3 panels.

The institutional investigation concluded the following about the other images:

- Figure 2D: difference analysis software indicated that lanes 1, 3 and 5 show a high proportion of similarity which may indicate duplication. Lanes 2, 4 and 6 show a high proportion of similarity and which may indicate duplication.
- Figure 4C: The bands in the hnRNP A2/B1 and hnRNPA1 panels are similar but not identical.

Given the extent of the above issues, the Editors have lost confidence in the paper.

